# Double deletion of tetraspanins CD9 and CD81 in mice leads to a syndrome resembling accelerated aging

**DOI:** 10.1038/s41598-018-23338-x

**Published:** 2018-03-23

**Authors:** Yingji Jin, Yoshito Takeda, Yasushi Kondo, Lokesh P. Tripathi, Sujin Kang, Hikari Takeshita, Hanako Kuhara, Yohei Maeda, Masayoshi Higashiguchi, Kotaro Miyake, Osamu Morimura, Taro Koba, Yoshitomo Hayama, Shohei Koyama, Kaori Nakanishi, Takeo Iwasaki, Satoshi Tetsumoto, Kazuyuki Tsujino, Muneyoshi Kuroyama, Kota Iwahori, Haruhiko Hirata, Takayuki Takimoto, Mayumi Suzuki, Izumi Nagatomo, Ken Sugimoto, Yuta Fujii, Hiroshi Kida, Kenji Mizuguchi, Mari Ito, Takashi Kijima, Hiromi Rakugi, Eisuke Mekada, Isao Tachibana, Atsushi Kumanogoh

**Affiliations:** 1 Department of Respiratory Medicine and Clinical Immunology, Osaka University Graduate School of Medicine, Suita, Osaka, Japan; 2 Sumitomo Dainippon Pharma Co., Ltd, Osaka, Japan; 3 National Institute of Biomedical Innovation, Health and Nutrition, Ibaraki, Osaka, Japan; 4 Department of Geriatric Medicine &, Osaka University Graduate School of Medicine, Suita, Osaka, Japan; 5 Department of Cell Biology, Research Institute for Microbial Diseases, Osaka University, Suita, Osaka, Japan

## Abstract

Chronic obstructive pulmonary disease (COPD) has been recently characterized as a disease of accelerated lung aging, but the mechanism remains unclear. Tetraspanins have emerged as key players in malignancy and inflammatory diseases. Here, we found that CD9/CD81 double knockout (DKO) mice with a COPD-like phenotype progressively developed a syndrome resembling human aging, including cataracts, hair loss, and atrophy of various organs, including thymus, muscle, and testis, resulting in shorter survival than wild-type (WT) mice. Consistent with this, DNA microarray analysis of DKO mouse lungs revealed differential expression of genes involved in cell death, inflammation, and the sirtuin-1 (SIRT1) pathway. Accordingly, expression of SIRT1 was reduced in DKO mouse lungs. Importantly, siRNA knockdown of CD9 and CD81 in lung epithelial cells additively decreased SIRT1 and Foxo3a expression, but reciprocally upregulated the expression of p21 and p53, leading to reduced cell proliferation and elevated apoptosis. Furthermore, deletion of these tetraspanins increased the expression of pro-inflammatory genes and IL-8. Hence, CD9 and CD81 might coordinately prevent senescence and inflammation, partly by maintaining SIRT1 expression. Altogether, CD9/CD81 DKO mice represent a novel model for both COPD and accelerated senescence.

## Introduction

Chronic obstructive pulmonary disorder (COPD) is a progressive disease state characterized by poorly reversible airflow limitation and an abnormal inflammatory response of the lungs to noxious particles, particularly cigarette smoke (CS)^1^. COPD is a growing cause of mortality and morbidity worldwide, and is expected to be the third leading cause of death by 2020^2^. In light of the considerable attention paid to the comorbidities of COPD, such as cardiovascular disease, diabetes mellitus, and osteoporosis, it is increasingly regarded as a systemic inflammatory lung disease^3,4^. Although the mechanisms underlying the relationship between COPD and these comorbidities remain unclear, the prevailing hypothesis is that a spill-over effect from the lung causes the extra-pulmonary comorbidities^5^: according to this theory, various inflammatory molecules such as CRP, IL-1β, and IL-6 secreted in the lung, spill out from the lung and induce systemic inflammation, as well as multi-organ disease. However, very few correlations between lung and serum markers have been observed, implying that a simple spill-over of mediators from the lung is not necessarily responsible for the systemic inflammation observed in COPD^6^. Given that the prevalence of COPD increases with age, that the abundance of alveolar senescent cells is elevated in the lungs of patients with COPD, and that COPD and aging share common mechanisms, COPD is considered to be a model for accelerated senescence of the lung, similar to other lifestyle-related diseases^7–9^. However, due to the complex nature of the mechanisms underlying COPD and aging, their precise interrelationship remains unclear.

Aging is a natural process characterized by progressive functional impairment and reduced capacity to respond appropriately to environmental stimuli and injury^10^. The hallmarks of aging include genomic instability, telomere attrition, epigenetic alterations, loss of proteostasis, deregulated nutrient sensing, mitochondrial dysfunction, cellular senescence, stem cell exhaustion, and altered intercellular communication^11^. Importantly, these mechanisms contribute to the pathogenesis of a variety of chronic diseases, including atherosclerosis, osteoporosis, cataracts, cancer, neurological diseases, and respiratory diseases^12,13^. Despite remarkable progress in the biology of aging over the past quarter century, the molecular mechanisms linking aging with age-related diseases have not yet been elucidated. However, the discovery of several aging models, such as Klotho, SAM, ATR, and SMP-30, has provided us with considerable new information regarding the pathogenesis of age-related diseases and potential therapeutic targets^14–17^. Among the key players in mammalian ageing, the sirtuins (SIRT1-SIRT7) are NAD+ dependent deacetylases that control a wide range of processes implicated in the regulation of homeostasis^18^. SIRT1, the best-characterized sirtuin in mammals, undoubtedly plays a key role in governing management of cellular stress management and ensuring a healthy lifespan^19^. SIRT1 expression is reportedly reduced in chronic inflammatory conditions, including aging^20^. Moreover, the activation or overexpression of SIRT1 increases lifespan in fly, yeast, worm, and mouse^21^. Importantly, SIRT1, whose expression is reduced in the lung of COPD patients, also plays pivotal roles in humans^22,23^. Because SIRT1 has critical effects in chronic inflammatory diseases, including cardiovascular disease and diabetes mellitus, considerable effort has been devoted to discovering pharmaceutical activators of SIRT1 for use in therapeutic applications^24^.

Tetraspanins are cell-surface proteins that span the membrane four times and are ubiquitously expressed in multiple organs^25–27^. A unique feature of tetraspanins is their propensity to interact with one another and with various other transmembrane molecules, including integrins and growth factor receptors, thereby acting as molecular organizers in tetraspanin-enriched microdomains. By organizing various functional molecules, tetraspanins are involved in a wide variety of biological processes, including cell migration, proliferation, survival, and morphogenesis, and thus influence immune diseases, infection, angiogenesis, and cancer metastasis^28^. CD9 and CD81, closely related tetraspanins, are expressed abundantly in the lung, and both CD9 knockout (KO) and CD81 KO mice exhibit quite similar phenotypes, such as infertility. Unexpectedly, younger CD9/CD81 double KO (DKO) mice develop COPD-like phenotypes^29,30^. Macrophages from DKO mice express elevated levels of MMP-9 production, probably due to disorganization of integrin-tetraspanin complexes in tetraspanin-enriched microdomains^30^. CD9 forms a complex with CD14, thereby stabilizing CD14/TLR4 complexes; consequently, CD9 KO mice exhibit enhanced macrophage-dominant inflammation and TNF-α production in the lungs after lipopolysaccharide stimulation^31^. Notably, CD9/CD81 DKO mice are more susceptible to cigarette-smoke-induced emphysema (manuscript in preparation). Our preliminary data suggest that levels of CD9 and CD81 are reduced in blood monocytes from COPD patients (paper in preparation). Considering that CD9/CD81 DKO mice develop not only emphysema but also some of the extra-pulmonary diseases seen in COPD patients, such as body weight loss and osteoporosis, we regard these DKO mice as a novel model for human COPD^32^. However, given that COPD is increasingly viewed as accelerated senescence of the lungs^8^, and that these tetraspanins have pleiotropic functions as molecular facilitators in a wide range of cells and tissues, we sought to explore the aging-like phenotype and its underlying mechanisms in this mouse model of COPD.

We found that CD9/CD81 DKO mice with the COPD-like phenotype progressively develop an accelerated aging syndrome, with symptoms including cataracts, osteoporosis, emphysema, and atrophy of the skin, muscle, and adipose tissue. In mechanistic terms, our findings revealed that double deletion of CD9/CD81 in epithelial cells downregulates the expression of SIRT1, thereby decreasing cell proliferation and augmenting inflammation. Thus, CD9/CD81 DKO mice could represent a unique model for COPD leading to accelerated senescence. Given that SIRT1 is considered a key molecule that protects against various lifestyle-related diseases and aging, the molecular organizers CD9 and CD81, which may maintain the expression of SIRT1 and other aging-related pathways, could serve as novel therapeutic targets, not only for COPD but also for aging more generally.

## Experimental Procedures

See the Supporting information for an expanded METHODS section.

### Mice

All animal experiments were approved by the Animal Care and Use Committee of Osaka University, and all of the animal procedures were performed in accordance with the Osaka University guidelines on animal care. CD9-KO, CD81-KO, and CD9/CD81-DKO mice were described previously^30,33,34^. These mice were backcrossed into the C57BL/6 J background for more than seven generations in a barrier facility, and all animal procedures were performed in accordance with the Osaka University guidelines on animal care. Genotyping of all breeding pairs was confirmed by PCR analysis. Eight- to 80-week (wk)-old DKO mice and wild-type (WT) littermates matched for age and sex were used.

### Cell culture and small interfering RNA (siRNA) transfection

A549 cells (human lung epithelial cell line) were cultured in DMEM containing 10% fetal bovine serum, 100 U/mL penicillin, and 100 μg/mL streptomycin. For siRNA transfection, the cells were transfected with an siRNA mixture against human CD9, CD81, or control random RNAs (B-Bridge International) using Lipofectamine RNAiMAX (Invitrogen). The cells were cultured for 2 days, and the gene-silencing effect of the siRNAs was assessed by immunoblotting with anti-CD9 and anti-CD81 monoclonal antibodies (Abs). In some experiments, the cells were trypsinized 2 days after transfection, re-cultured in DMEM overnight, and then stimulated with 50 ng/mL TNF-α. The cells were harvested after a further 24-h incubation. Alternatively, 5 µM SRT1720 (Cayman Chemical) was added to the culture at 4 h after CD9 and CD81 siRNA transfection, and the cells were harvested 2 days after transfection. Concentrations of IL-8 in culture supernatants were measured by enzyme-linked immunosorbent assay using Quantikine (R&D Systems).

### Proliferation and apoptosis assays

The number of viable cells was determined using a Cell Counting Kit-8 (Dojindo). Cells (3.0 × 10^4^), transfected with siRNA against human CD9 and CD81 or control RNAs, were seeded on 96-well plates and incubated overnight. After further incubation for 2 days, the kit reagent WST-8 was added to the medium, and the cells were incubated for 1 h. The absorbance of samples (450 nm) was determined using a scanning multi-well spectrophotometer. For the apoptosis assay, 3.0 × 10^4^ cells were seeded on 96-well plates and incubated overnight. After further incubation for 2 days, quantities of histone-associated DNA fragments in cell lysates were measured using Cell Death Detection ELISA PLUS (Roche).

### Immunoblotting

Tissues or cells were lysed in a lysis buffer containing 25 mM Tris-HCl (pH 7.6), 150 mM NaCl, 1% NP-40, 1% sodium deoxycholate, and 0.1% SDS supplemented with or without Halt Protease and Phosphatase Inhibitor Cocktail (ThermoFisher Scientific). Lysates containing equal amounts of protein were separated by SDS-PAGE, transferred to PVDF membranes, and probed with primary Abs followed by peroxidase-conjugated secondary Abs. The following primary Abs were used: mouse anti-human CD9 (MM2/57; Invitrogen), mouse anti-human CD81 (JS64; BECKMAN COULTER), rat anti-mouse CD9 (KMC8; BD Bioscience), hamster anti-mouse CD81 (Eat2; AbD SeroTec), mouse anti-human SIRT1 (1F3; Cell Signaling Technology [CST]), mouse anti-mouse SIRT1 (19A7AB4; Abcam), rabbit anti-human SIRT6 (EPR5079(N); Abcam), rabbit anti-mouse SIRT6 (D8D12; CST), rabbit anti-FOXO3a (CST), mouse anti-p53 (1C12; CST), rabbit anti-p21 (12D1; CST), rabbit anti-p21 (EPR18021; Abcam), mouse anti-p16 (D25; CST), rabbit anti-Klotho (Abcam), mouse anti-WRN (8H3; CST), rabbit anti-ATR (CST), NF-κB (E379; Abcam), rabbit anti-phospho-NF-κB p65 (93H1; CST), rabbit anti-acetyl-NF-κB p65 (D2S3J; CST), rabbit anti-IκB (CST), and rabbit anti-β-actin (13E5; CST). Immunoreactive signals were visualized using SuperSignal West Pico Chemiluminescent Substrate (ThermoFisher Scientific). For densitometry, blots were analyzed on a LAS-3000 Imager (Fujifilm) or Amersham Imager 600 (GE Healthcare).

### Flow cytometry analysis

Isolated cells from mouse spleen were incubated with anti-CD16/CD32 mAb (2.4G2, BD Pharmingen) to block the Fc-receptor and labeled with the following Abs (purchased from BD-Pharmingen) for flow cytometry analysis: rat anti-mouse CD4-FITC (GK1.5), rat anti-mouse CD8-PE (53-6.7), rat anti-CD11b-PE (M1/70), rat anti-mouse CD19-APC (1D3), rat anti-mouse CD21-FITC (7G6), rat anti-mouse CD23-PE (B3B4), mouse anti-mouse IgD-FITC (AMS9.1), goat anti-Ig-PE, rat anti-mouse B220-APC or B220-FITC (RA3-6B2), and anti-DX5 (Ha1/29). Stained cells were analyzed on a BD FACSCanto II (BD Biosciences).

### Histology and histomorphometric analysis of the lung

Tissue samples were excised and fixed in 10% buffered neutral formalin and embedded in paraffin. The sections were stained with hematoxylin-eosin (HE), periodic acid-Schiff (PAS), or toluidine blue. In some experiments, tissues were fixed by perfusion of the fixative. Lungs were inflated to 25 cm of water pressure with 10% buffered neutral formalin via an intratracheal cannula and embedded in paraffin.

### Immunofluorescence

Cells were cultured in glass-bottom plates, fixed in 3% paraformaldehyde, and then permeabilized with Tris-buffered saline containing 0.3% Triton X-100. Nonspecific recognition was blocked with Blocking One (Nacalai Tesque). The permeabilized cells were incubated with 1 µg/mL primary Ab overnight at 4 °C and subsequently incubated with Alexa Fluor 488-conjugated goat anti-mouse, goat anti-rabbit, or goat anti-rat Abs (Molecular Probes). Nuclei were visualized using DAPI (Molecular Probes). For actin filament staining, rhodamine phalloidin (Molecular Probes) was used. Immunofluorescence images were obtained using the Leica TCS SPE microscope system (Leica Microsystems). The following primary Abs were used: mouse anti-human CD9 (MM2/57; Invitrogen), mouse anti-human CD81 (JS64; BECKMAN COULTER), mouse anti-human SIRT1 (1F3; CST), rabbit anti-cleaved caspase-3 (CST), and rabbit anti-Ki-67 (D3B5; CST).

### Senescence-associated β-galactosidase (SA-β-Gal) staining

Cytochemical staining for SA-β-Gal activity was performed using a Senescence Detection Kit (BioVision). Cells (1.0 × 10^5^ or 5.0 × 10^5^), transfected with siRNA against human CD9 and CD81 or control RNAs, were seeded on 24-well plates. After incubation for 2 days, the cells were fixed, and a staining solution containing X-Gal was added to the fixed cells and incubated for 20 h at 37 °C. The proportion of cells positive for SA-β-Gal activity was determined by counting the number of blue cells in four independent microscope fields.

### Aging score

Aging scores were assessed as described previously with minor modifications^15^. Specifically, the senescence scores of mice were graded according to their behavior and the appearance of the skin and spine.

### DNA microarrays and PCR arrays

Lungs removed from mice (WT [n = 4] and CD9/CD81 DKO [n = 3] at 15 wk of age or WT [n = 1] and CD9/CD81 DKO [n = 1] at 12 wk of age) were immersed in RNAlater solution (Invitrogen) and stored at −80 °C until RNA extraction. The lungs were transferred into TRIzol reagent (Invitrogen) and homogenized. Cells transfected with siRNA against human CD9 and CD81 (n = 3) or control RNAs (n = 3) were harvested and lysed in RLT reagent (Invitrogen). Total RNA was extracted from the lysates using the RNeasy mini kit (QIAGEN). After reverse transcription, cDNA was hybridized using Agilent SurePrint G3 Mouse or Human GE Microarrays (Agilent Technologies) according to the protocol specified in the Agilent Gene Expression Hybridization Kit (Agilent Technologies). Microarray signal intensity was normalized using the RMA algorithm implemented in GeneSpring GX ver.12 (Agilent Technologies). Alternatively, gene expression profiles of inflammatory cytokines in RNA extracted from A549 cells treated or not treated with TNF-α were obtained by RT-PCR using TaqMan Array Plates; Human cytokine network (Applied Biosystems) on a 7900HT Fast Real Time PCR System (Applied Biosystems).

### DNA microarray analysis and bioinformatics

To identify biologically relevant molecular networks and pathways in gene expression data from mice lungs or epithelial cells, three distinct bioinformatic pathway analysis tools were used: Ingenuity Pathways Analysis (IPA)^35^, TargetMine^36^, and KeyMolnet^37^.

### Statistical analysis

**Figure 1:**
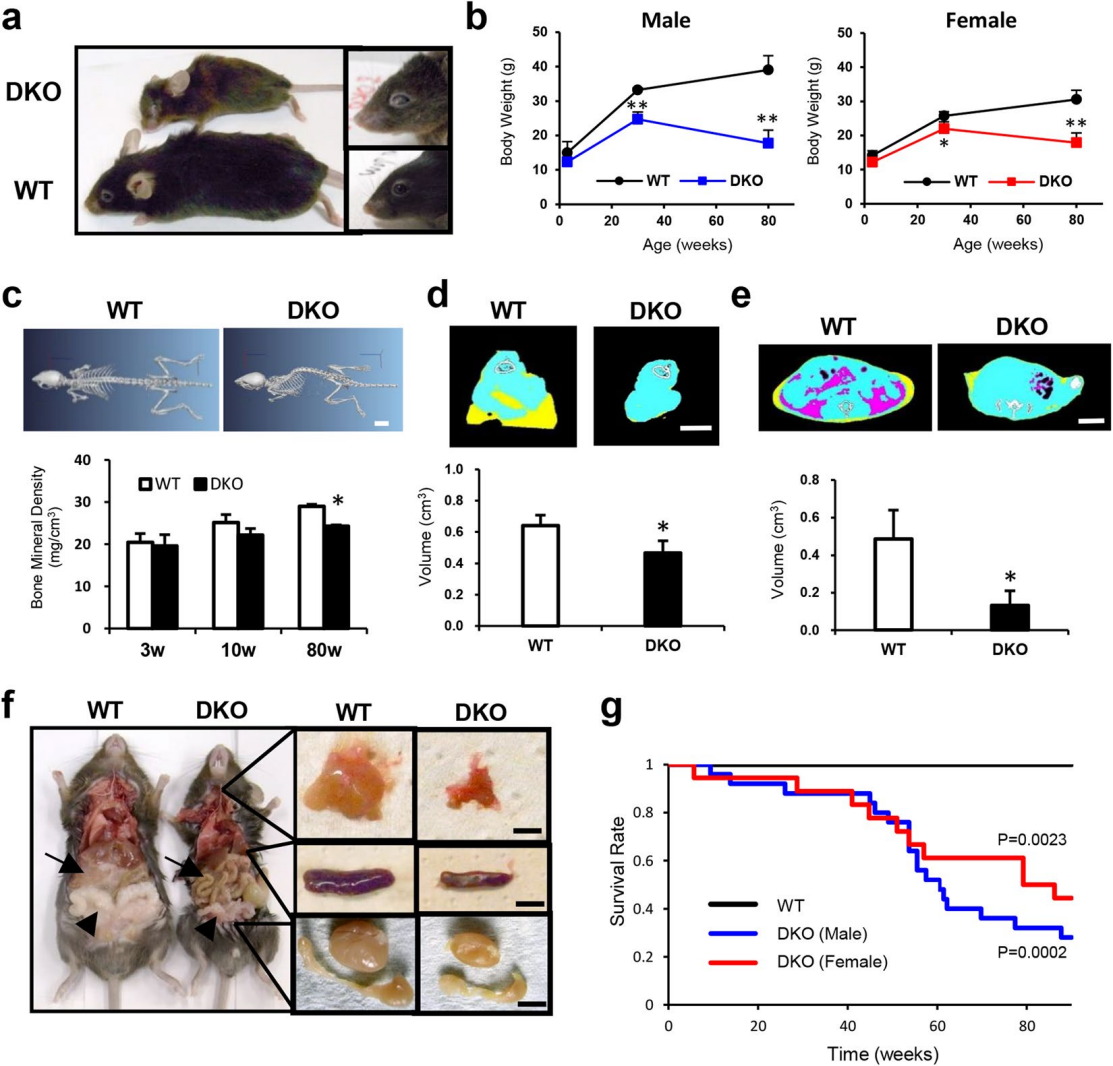
CD9/CD81 DKO mice progressively exhibit aging phenotype with shorter survival. (**a**) Representative photo from WT mouse (lower) and DKO mice (upper) at 80 weeks of age, Whole body (left) and eyes (right) from each group. (**b**) Body weight (left: male; n = 3–6, right: female; n = 3–5). DKO mice exhibit infertility, as described previously. (**c**) Representative CT images of the body trunk of WT and DKO mice at 80 weeks of age. Note that DKO mice exhibit severe kyphosis. Bone mineral density (BMD) decreased with age (n = 2–4). (**d**,**e**) Quantitation of volume of muscle (n = 4–5) and adipose tissue by micro-CT (n = 2–4). Note the reduction in volume of muscle and adipose. Yellow: subcutaneous fat, Pink: visceral fat, Blue: muscle. (**f**) Gross necropsy of euthanized moribund mice revealed a cachectic appearance. Isolated thymus (upper), spleen (middle), and testis were smaller in DKO mice (left: WT, right: DKO). (**g**) Comparison of lifespans. Survival was analyzed by the Kaplan-Meier method and log-rank test. WT: male (n = 14), female (n = 24), DKO: male (n = 25), female (n = 19) Note that DKO mice of both sexes exhibit shorter survival. Bars represent means ± SD; *P < 0.05, **P < 0.01 versus WT. Scale bar, 10 mm for (**c**), 30 mm for (**d**) and (**e**) and 5 mm for (**f**).

All numerical results are expressed as means ± standard deviation. Separate animals were examined in experiments containing multiple time points in Fig. 1b,c. Statistical analysis of mice survival was performed by the Kaplan-Meier method and log-rank test using GraphPad PRISM ver.6. Except for the survival experiments, statistical significance was evaluated using Student’s t test, and P-values < 0.05 were considered statistically significant.

## Results

### CD9/CD81 DKO mice progressively exhibit multiple aging phenotypes with shorter survival

At 80 weeks of age, CD9/CD81 DKO mice were smaller and had less hair of a brownish color than WT mice, although DKO and WT mice could not be distinguished at 3 wk of age (Fig. 1a). In addition, the aged DKO mice had cloudy eyes. Although aged DKO and WT mice consumed comparable amounts of food, both male and female DKO mice progressively lost body weight (Fig. 1b and data not shown). However, neither CD9 KO mice nor CD81 KO mice exhibited such aging-related phenotypes (data not shown). Moreover, DKO mice developed progressive kyphosis and decreased bone mineral density (Fig. 1c). Muscle and visceral adipose tissue were significantly reduced in volume, as determined by CT quantitation (Fig. 1d,e). Consistent with this cachectic appearance, at necropsy, we observed a decrease in adipose tissue and diminution of the thymus and spleen, as well male reproductive organs such as testis and seminal vesicles (Fig. 1f). Consequently, DKO mice had remarkably shorter survival than WT mice (Fig. 1g); the cause of death was not identified, but the frequency of malignant tumors was indistinguishable between the two groups. Together, these findings indicate that aged CD9/CD81 DKO mice exhibited a variety of accelerated aging phenotypes.

### CD9/CD81 DKO mice exhibit multiple histological phenotypes of aging

**Figure 2:**
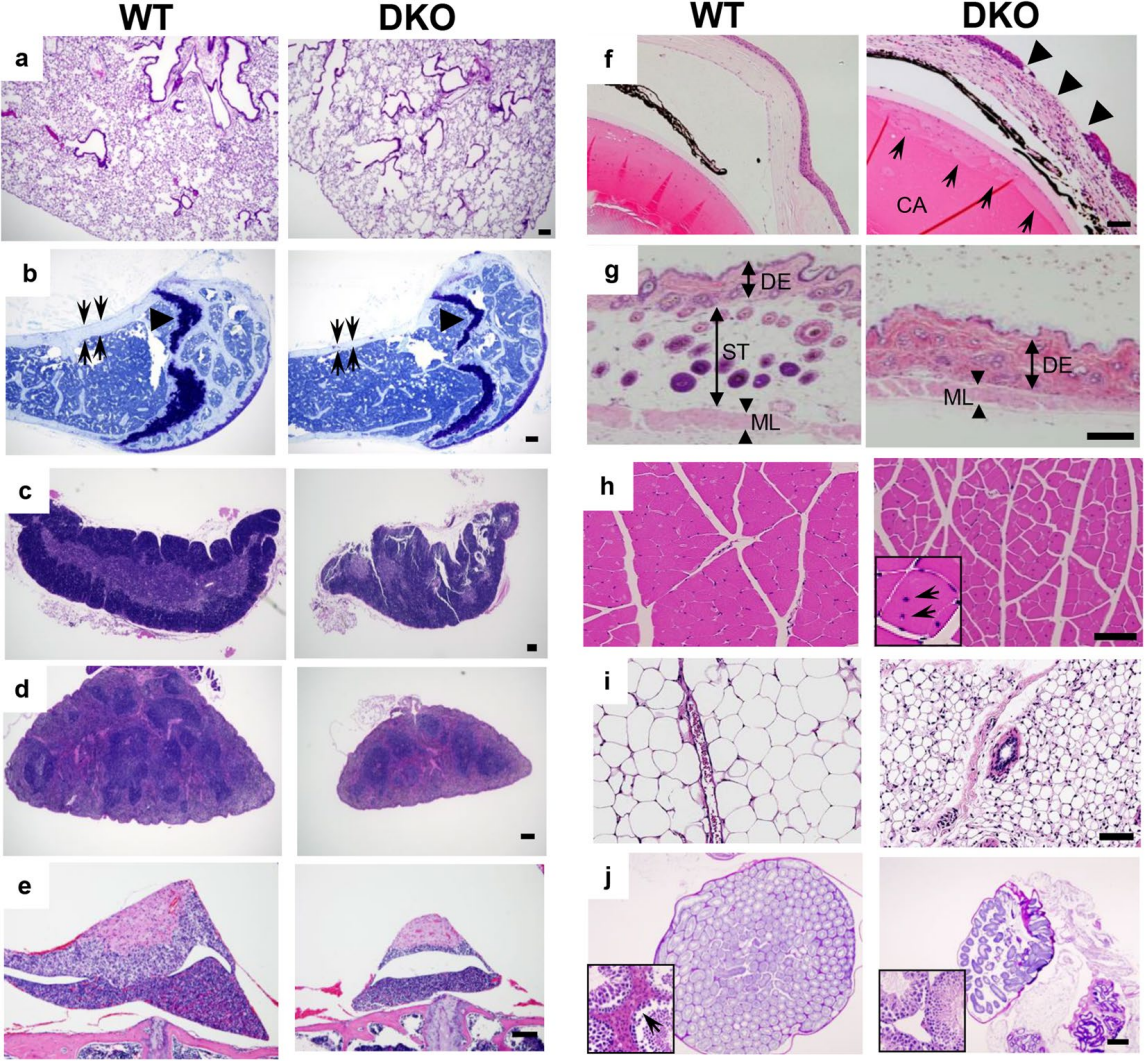
CD9/CD81 DKO mice exhibit multiple histological aging phenotypes. (**a**–**j**) Histological sections from lungs (**a**), femur (**b**), thymus (**c**), spleen (**d**), pituitary (**e**), eye (**f**), skin (**g**), muscle (**h**), adipose (**i**), and testis (**j**). (**a**) Emphysematous lungs in DKO mice. (**b**) Note that the cortex (arrow) and growth plate (arrowhead) were much thinner in DKO. (**c**,**d**,**e**) Atrophy in the thymus, spleen, and pituitary in DKO mice. (**f**) Keratitis (arrows) and cataracts (CA) were remarkable in DKO mice. (**g**) Less subcutaneous fat and fewer hair follicles were observed in DKO mice. DE: dermis, ST: subcutaneous tissue, ML: muscle layer. (**h**) DKO mice exhibited diffusely atrophic myofibers, along with the central nuclei (arrows, inset). (**j**) Testis in DKO mice were more atrophic, and the numbers of both Sertoli cells and Leydig cells (inset) were severely diminished. All sections were stained with hematoxylin-eosin (HE), except testis (**j**), which was stained with periodic acid-Schiff (PAS), and femur (**b**), which was stained with toluidine blue. Data are representative of three independent studies with similar results. Scale bar, 0.5 mm for (**g**) and 100 μm for (**a**–**f**) and (**h**–**j**).

Histological examination revealed that DKO mice developed emphysema and osteoporosis at 80 wk (Fig. 2a,b). Consistent with their macroscopic appearance, DKO mice exhibited atrophic thymus and spleen (Fig. 2c,d, and Supplemental Table 1). Moreover, the pituitary gland was atrophic, and keratitis and cataracts developed in DKO mice at 80 wk of age (Fig. 2e,f, Supplemental Table 1). At the same time, the number of hair follicles and amount of subcutaneous fat were dramatically reduced (Fig. 2g). Furthermore, aged DKO mice exhibited atrophy in muscle, adipose tissue, and testis; none of these phenotypes were observed in younger DKO mice (Fig. 2h–j and data not shown). Notably, a diffuse decrease in myofiber size, accompanied by central and multinucleated nuclei, was observed in muscle from DKO mice (Fig. 2h and inset). In addition, we observed atrophy in the submandibular gland, pancreatic acinus, epididymis, and prostate, and the numbers of vacuoles and enlarged glial cells in the cerebrum were elevated (Supplemental Fig. 1). Although no histological differences could be detected in the ovary, the atrophy of the testis of DKO mice was remarkable. For example, the numbers of seminiferous tubules and Leydig cells, which support spermatogenesis and testosterone production, respectively, were both decreased (Fig. 2j). Conversely, no significant difference was observed in other organs such as the heart, kidney, liver, gastrointestinal tract, and aorta (Supplemental Table 1 and data not shown). Hematological and serological tests revealed that the levels of creatinine and phosphate were indistinguishable, except for a minimal decrease in cholesterol (Supplemental Table 2). Moreover, by immunostaining for p21, a senescence associated molecule, the number of p21-positive cells increased in lung sections in DKO mice (Supplemental Fig. 2). Combined with the macroscopic findings, DKO mice progressively developed accelerated senescence with multiple aging phenotypes.

### CD9/CD81 DKO mice exhibit reduced physical activity and develop immunosenescence

**Figure 3:**
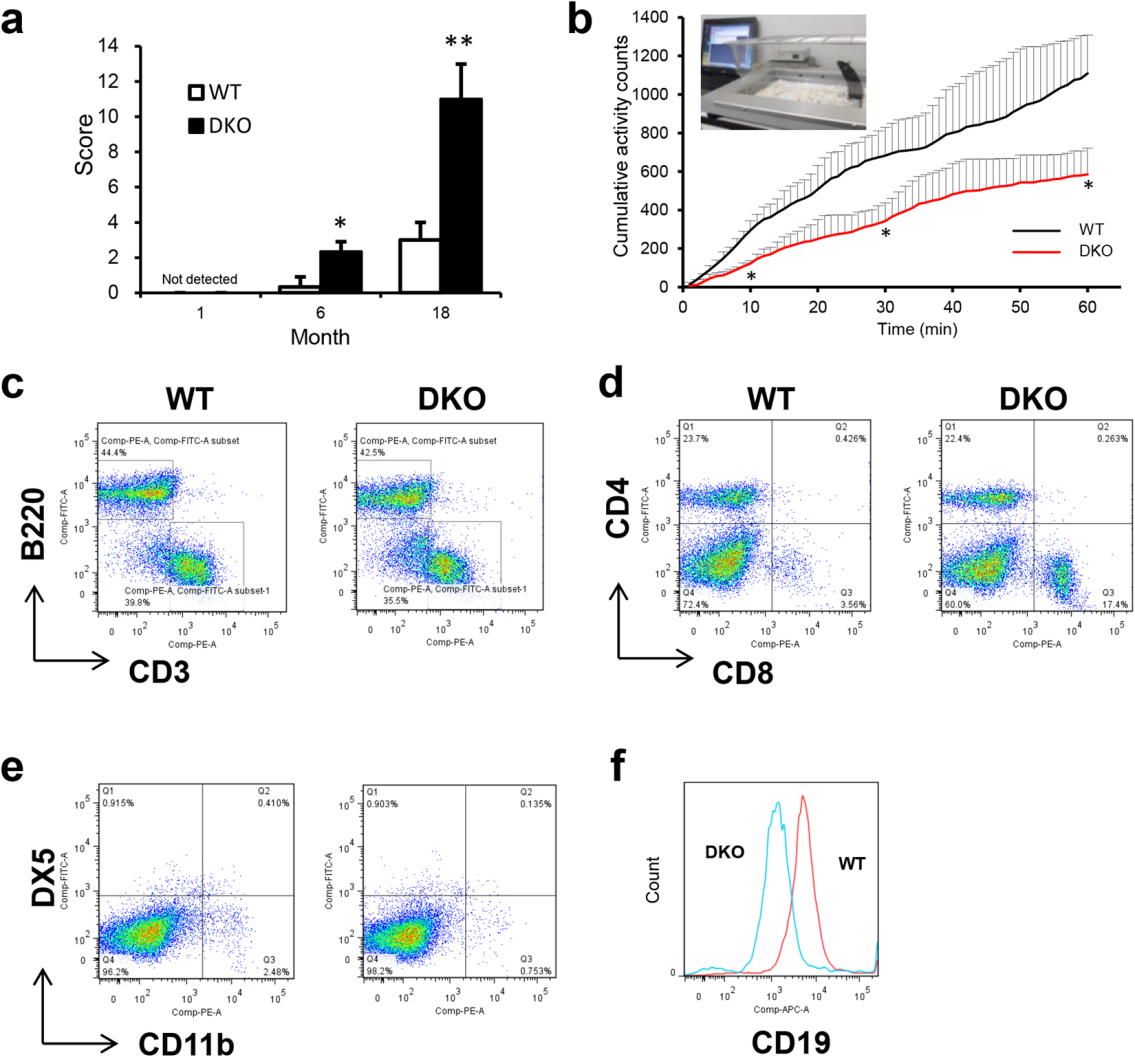
CD9/CD81 DKO mice exhibit reduced physical activity and develop immunosenescence. (**a**) Aging scores calculated for WT and DKO mice at 1, 6, and 18 months of age (n = 3). Note that DKO mice exhibited a significantly elevated aging score even at 6 months of age. (**b**) Mean integrated spontaneous locomotor activity evaluated using the LOCOMO sensor system (n = 3). (**c**–**f**) FACS data with splenocytes from WT and DKO mice at 70 weeks of age. B220: B cells, Thy1.2: T cells, CD4 and CD8: T helper cell subset, CD11b: neutrophils, DX5: NK cells, CD19: B cell subset. Bars represent means ± SD; *P < 0.05, **P < 0.01 versus WT.

To evaluate functional impairment, we calculated an aging score, as described previously^15^. Although younger DKO mice at 1 month of age did not exhibit any differences, mice at 18 months of age had an elevated aging score compared to WT mice, which was true even at 6 months of age (Fig. 3a and Supplemental Fig. 2a). In addition, locomotive activity was markedly reduced in DKO mice (Fig. 3b). Consistent with the histological findings such as muscle atrophy and emphysema (Fig. 2), DKO mice exhibited reductions in both grip strength and respiratory function (Supplemental Fig. 3b,c).

Because macroscopic and microscopic findings revealed significant atrophy in the thymus and spleen, we further examined immunological function in aged mice. Consistent with the histological finding of spleen atrophy, the total number of spleen cells in DKO mice was 3.6-fold lower than in WT mice. Moreover, Although the CD4/CD8 ratio, one of markers in immunosenesence, was not altered in younger mice, it was reduced in aged DKO mice in comparison with WT mice (Fig. 3d and data not shown)^38^. On the other hand, T cells, B cells and NK cells were not altered in DKO mice compared to those in WT mice, although neutrophils were slightly decreased in DKO mice (Fig. 3c,e). As reported in CD81 KO mice^34^, the expression of CD19 in B cells was markedly reduced in DKO mice (Fig. 3f). Taken together, these observations indicate that DKO mice exhibited reduced physical activities and developed immunosenescence.

### Comprehensive DNA microarray analysis reveals the underlying mechanisms of the aging phenotypes

**Figure 4:**
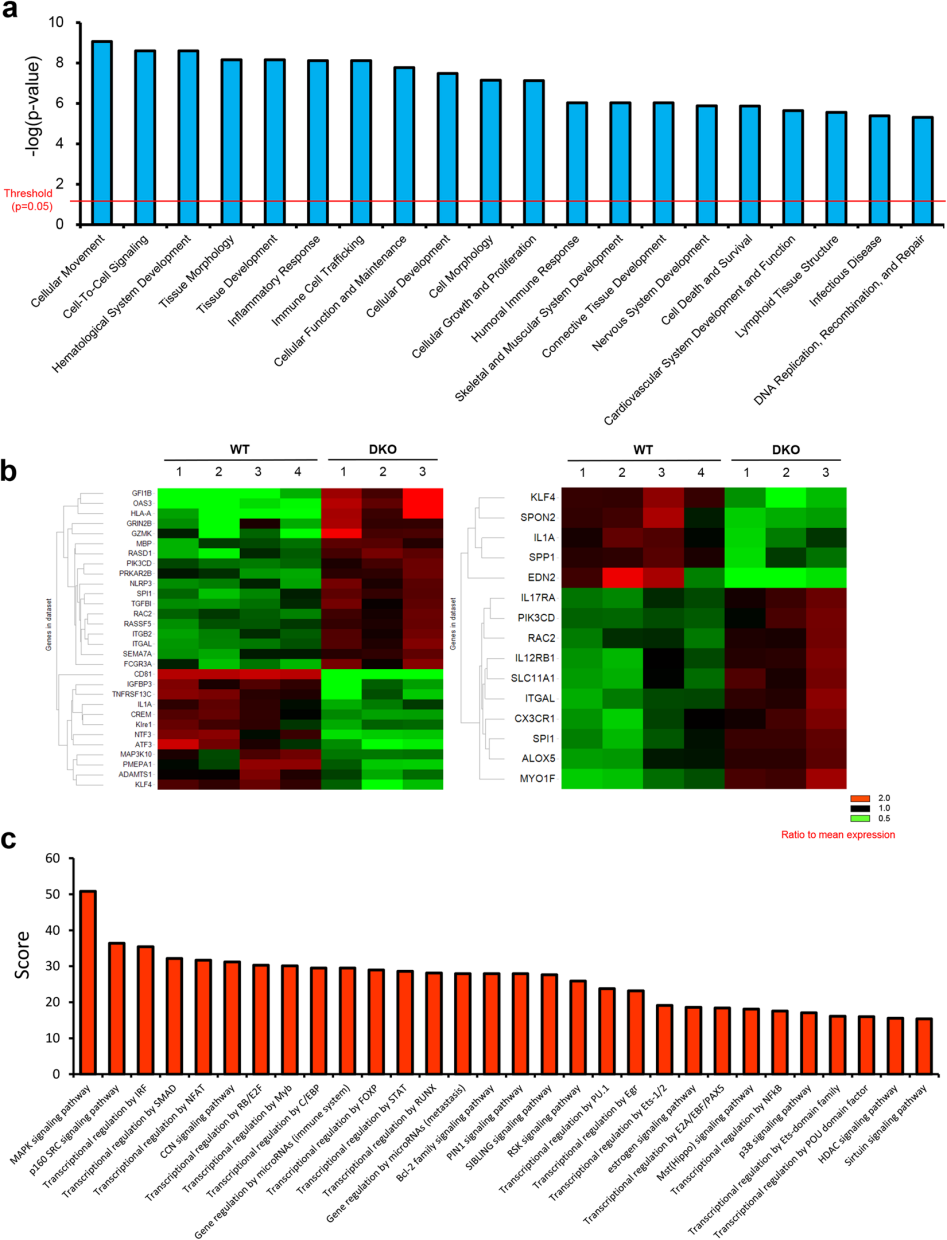
Comprehensive DNA microarray analysis of the lung. (**a**) Differentially expressed genes between WT (n = 4) and CD9/CD81 DKO (n = 3) lungs were analyzed using IPA, and the results shown are the top 20 categories of differentially expressed genes in DKO lungs. (Threshold: *P* < 0.05) (**b**) Differentially expressed genes in the enriched gene category “cell death” (left) and “inflammatory response” (right) are shown. The heat map indicates that genes promoting cell death or inflammatory response were upregulated in DKO lungs. Red: higher than mean value, Green: lower than mean value. (**c**) Molecular pathways were analyzed using KeyMolnet software. Results shown are the top 30 molecular pathways associated with genes differentially expressed in DKO lungs.

To explore the mechanisms of the aging phenotypes observed in CD9/CD81 DKO mice, we performed DNA microarrays. Consistent with the aging phenotypes, IPA revealed that genes associated with inflammation, cell death, cardiovascular disorder, and skeletal muscular disorder were highly enriched among the genes differentially expressed in DKO mice (Fig. 4a). Moreover, differentially expressed genes in the categories “cell death” and “inflammatory response” were enriched in DKO lungs, as revealed by heat map analysis (Fig. 4b). The biological processes identified by TargetMine, which can convert mouse data to human orthologs, were similar to those highlighted by IPA, and included inflammation and cell death (Supplemental Fig. 4b,c). These data suggest that the aging phenotypes observed in DKO mice might recapitulate the aging process in humans. Using the KeyMolnet software, which identifies molecular pathways^37^, we found that the top 30 pathways associated with the differentially expressed genes in DKO lungs included the sirtuin pathway (Fig. 4c). Indeed, network analysis revealed relevant interactions between these tetraspanins and aging-related molecules, including SIRT1 (Supplemental Fig. 5).

### Tetraspanins CD9 and CD81 coordinately maintain the expression of SIRT1

**Figure 5:**
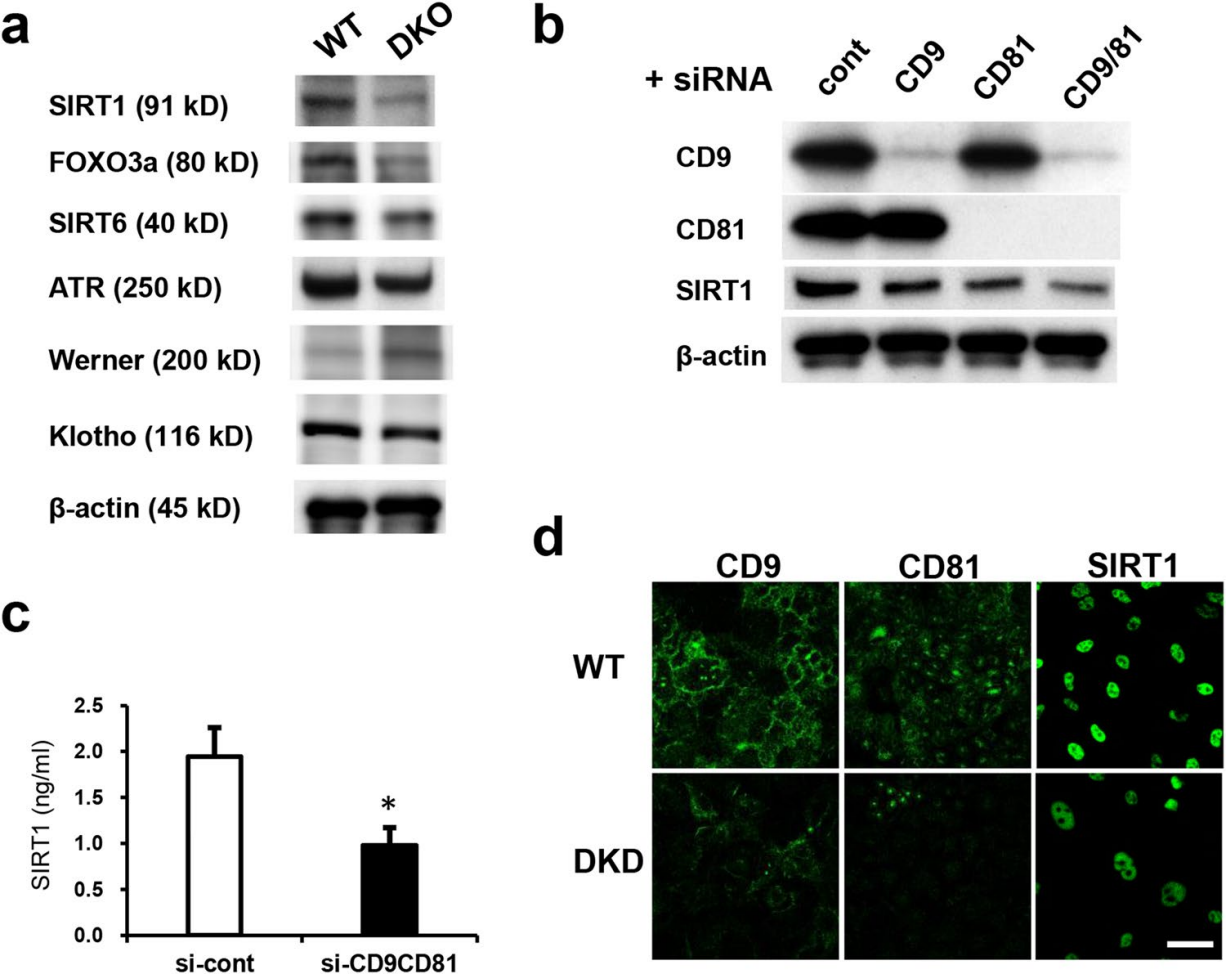
Double deletion of CD9/CD81 results in reduced SIRT1 expression. (**a**) Immunoblot of the lungs for major aging molecules. Notably, expression of SIRT1, but not that of SIRT6, Klotho, WRN, and ATR, was reduced in DKO lungs. (**b**) Knockdown of CD9 and CD81 expression with siRNA in epithelial cells additively downregulated the expression of SIRT1. (**c**) SIRT1 expression by ELISA (n = 4). (**d**) SIRT1 expression by immunocytochemistry. Bars represent means ± SD; *P < 0.05 versus si-cont. Scale bar, 50 µm for (**d**).

Consistent with the DNA microarray data, expression of SIRT1 and its downstream target Foxo3a was reduced in the lungs of CD9/CD81 DKO mice, whereas the levels of other aging molecules such as SIRT6, Klotho, WRN (Werner syndrome ATP-dependent helicase), and ATR (ataxia telangiectasia and Rad3-related protein) were unaltered (Fig. 5a and data not shown). Given that SIRT1 in epithelial cells is regarded as a key molecule in COPD^23^, and that genes associated with the SIRT1 pathway were differentially regulated in DKO lungs (Fig. 4c), we focused our analysis on the SIRT1 pathway. Notably, knockdown of CD9 and CD81 in epithelial cells additively downregulated the expression of SIRT1, whereas knockdown of CD151 did not (Fig. 5b and data not shown). The reduced expression of SIRT1 was further verified by immunocytochemistry and ELISA (Fig. 5c,d). Collectively, these findings indicated that the closely related tetraspanins CD9 and CD81 maintain the expression of SIRT1 in lung and epithelial cells.

### Double deletion of CD9/CD81 increases apoptosis and decreases cell proliferation

**Figure 6:**
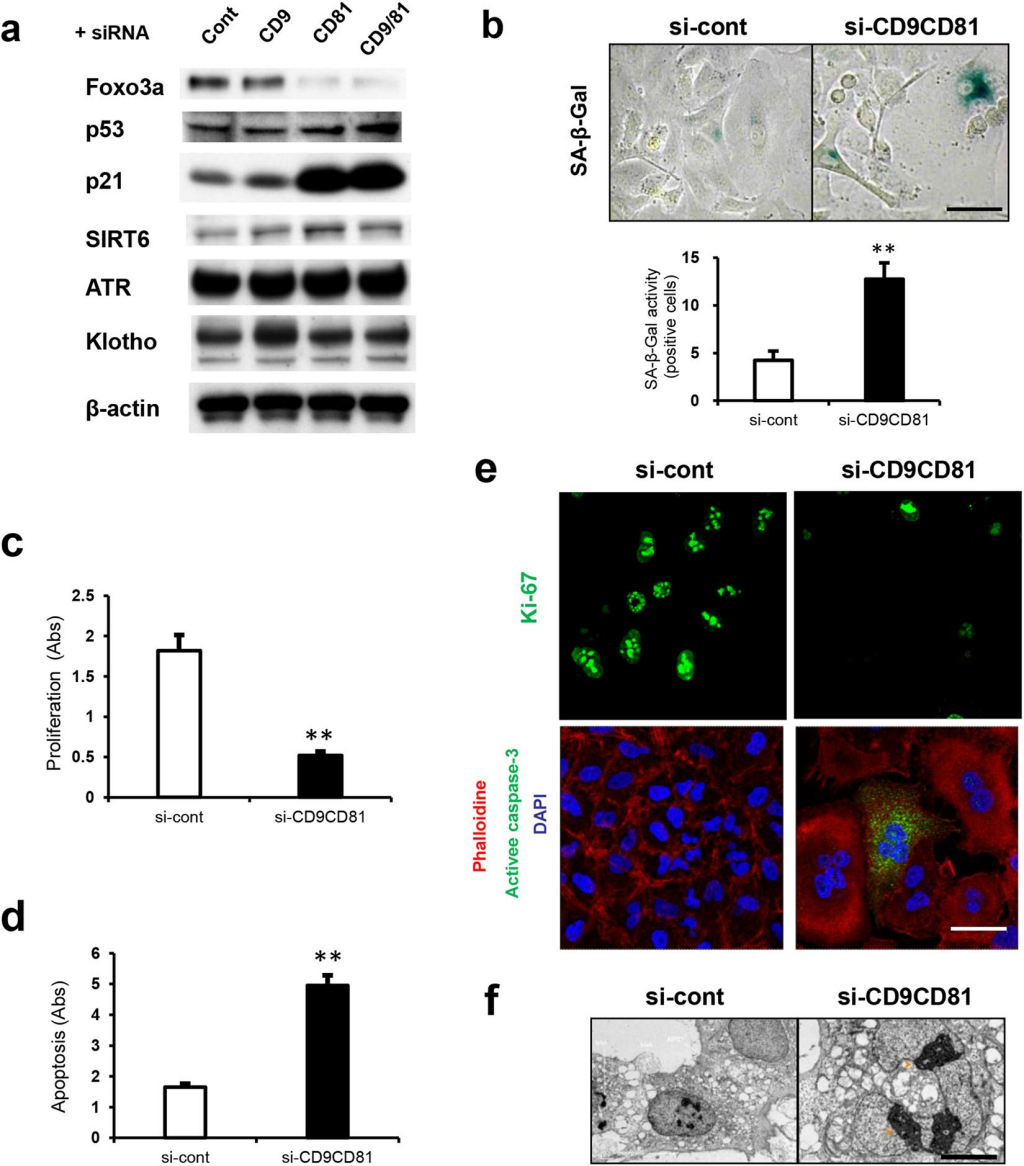
Double knockdown of CD9/CD81 results in reduced SIRT1 expression, thereby increasing apoptosis and decreasing cell proliferation. (**a**) siRNA knockdown of CD9 and CD81 in epithelial cells downregulated the expression of Foxo3a, but not that of Klotho, ATR and SIRT6. Reciprocally, expression of p53 and p21 was upregulated. (**b**) SA-β-gal staining of DKD epithelial cells (n = 4). (**c**) Proliferation assay (n = 4). (**d**) Apoptosis assay (n = 4). (**e**) ICC image of DKD epithelial cells. (**f**) Electron microscopy of knockdown epithelial cells. Scale bar, 50 µm for (**b**) and (**e**) and 5 µm for (**f**). Bars represent means ± SD; **P < 0.01 versus si-cont.

In accordance with the DNA microarray analysis of the lungs, analysis of DKO epithelial cells also revealed similar aging phenotypes, with differential regulation of genes associated with cell survival, cellular proliferation, cardiovascular disorder, cell cycle and immune responses (Supplemental Fig. 6). After knocking down CD9/CD81 in epithelial cells, expression of ATR and Klotho was unchanged, but expression of Foxo3a was reduced (Fig. 6a). By contrast, cell-cycle regulators such as p53 and p21 were upregulated. Double knock-down (DKD) of CD9/CD81 in epithelial cells resulted in cells with a large flattened morphology, and the proportion of SA-β-Gal-positive (i.e., senescent) cells increased (Fig. 6b). Importantly, elevated apoptosis and reduced cell proliferation are considered to be major mechanisms underlying both COPD and aging. Indeed, DKD epithelial cells exhibited reduced proliferation and elevated apoptosis (Fig. 6c,d). The proportion of Ki-67-positive (i.e., proliferating) cells decreased, whereas the proportion of active caspase-3-positive (i.e., apoptotic) cells increased (Fig. 6e). Moreover, DKD epithelial cells exhibited not only fragmented nuclei but also an elevated abundance of lysosomes (Fig. 6f and data not shown). Importantly, SRT1720, a SIRT1 activator, rescued the reduced expression of Foxo3a and reciprocally downregulated the expression of p21, suggesting that some of the observed aging phenotypes were partly dependent on SIRT1 activity (Supplemental Fig. 7). Together, these findings show that double knockdown of CD9/CD81 in epithelial cells downregulated the SIRT1 pathway, thereby affecting cell survival, proliferation, and senescence.

### Double deletion of CD9/CD81 induces inflammation

**Figure 7:**
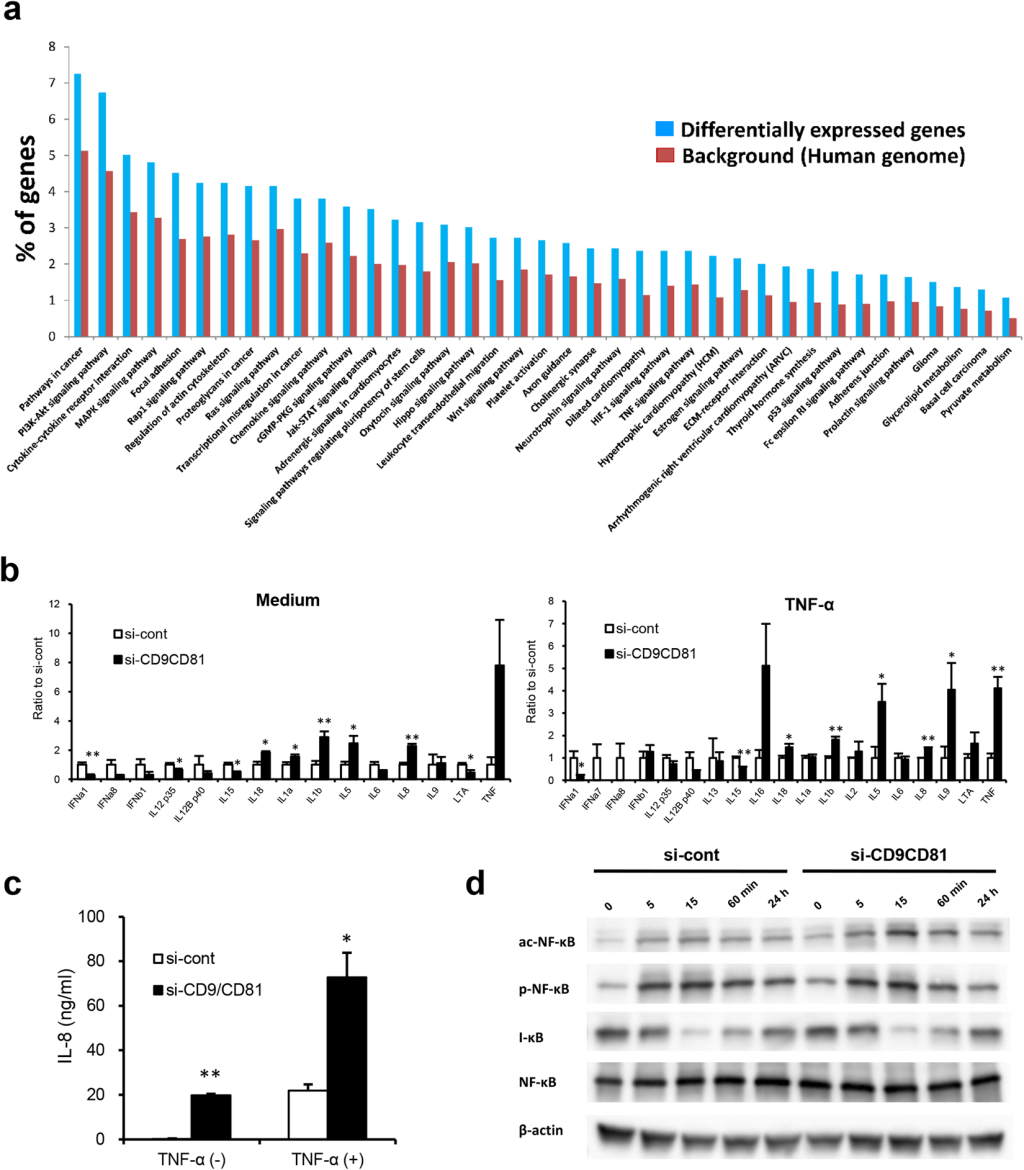
Double deletion of CD9/CD81 increases inflammation. (**a**) DNA microarray analysis of CD9/CD81 DKD epithelial cells using TargetMine revealed enriched KEGG pathways, including inflammation-associated and proliferative pathways (n = 3). (**b**) Real-time PCR analysis of epithelial cells using a TaqMan Array Plate (Human Cytokine Network) revealed that CD9/CD81 DKD upregulated the gene expression of inflammatory cytokines such as IL-1β, IL-8, and TNF-α (n = 3). (**c**) CD9/CD81 DKD epithelial cells exhibited elevated IL-8 production (n = 3). (**d**) Kinetics of NF-κB and acetylated or phosphorylated NF-κB induced by TNF-α in A549 epithelial cells. DKD of CD9 and CD81 increased the levels of acetylated NF-κB. Bars represent means ± SD; *P < 0.05, **P < 0.01 versus si-cont.

Along with senescence, another important aspect of aging is inflammation (also called inflammaging). Our DNA microarray analysis indicated that the expression profile of genes related to cellular movement and immune response of CD9/CD81 DKD epithelial cells was similar to that of DKO lungs (Supplemental Fig. 6). Moreover, among the KEGG pathways, inflammatory pathways such as chemokine and TNF signaling were highly ranked along with cell death (Fig. 7a). Hence, to determine the inflammatory response *in vitro*, we analyzed inflammation in DKD epithelial cells. Quantitative PCR analysis revealed that a variety of cytokines, including IL-8, IL-1β, and TNF-α, were upregulated by TNF-α stimulation (Fig. 7b). Upon TNF-α stimulation, IL-8 production was significantly elevated in DKD epithelial cells (Fig. 7c). Notably, while acetylation of NF-κB was increased by TNF-α stimulation, levels of phosphorylated NF-κB, I-κB, and NF-κB were not altered in DKD epithelial cells, suggesting that the inflammatory phenotypes were also partially dependent on the SIRT1 pathway (Fig. 7d). Thus, the tetraspanins CD9 and CD81 maintained the expression of SIRT1, thereby preventing senescence and inflammaging.

## Discussion

In this study, we revealed that (1) double deletion of the related tetraspanins CD9 and CD81 in mice caused progressive multiple aging phenotypes and a shortened life span, and (2) CD9 and CD81 play coordinate roles in maintaining SIRT1 expression, thereby facilitating anti-aging and anti-inflammatory activity.

### From COPD model to accelerated senescence: a novel aging model

Based on our previous work, we regarded CD9/CD81 DKO mice as a COPD-like model. Surprisingly, aged CD9/CD81 DKO mice developed not only emphysema but also non-pulmonary comorbidities such as osteoporosis and body weight loss, eventually leading to multiple aging phenotypes. Given that senescence in epithelial and endothelial cells is elevated in patients with COPD^7^, COPD has increasingly been viewed as an accelerated senescence of the lung. Given this background, CD9/CD81 DKO mice could be regarded as a novel model for both COPD and aging.

To date, several aging models with distinct features have been reported^39^. Among them, Klotho-deficient mice exhibit multiple aging phenotypes including osteoporosis, emphysema, arteriosclerosis, and atrophy in major organs including the sex organs, skin, and pituitary, whereas overexpression of Klotho extends the lifespan of mice^14,40^. Notably, the discovery of the Klotho gene has not only advanced our understanding of the aging process, but also the development of therapeutic applications. Although CD9/CD81 DKO mice have a similar phenotype to Klotho mice, a few major differences should be noted. First, although Klotho is specifically expressed in the kidney and brain, both CD9 and CD81 are ubiquitously expressed throughout whole body, albeit to varying degrees^26^. Second, α-Klotho is a multifunctional protein similar to a hormone, and regulates the metabolism of phosphates, calcium, and vitamin D. Consistent with this hormone-like role, Klotho mice develop aging phenotypes such as emphysema and osteoporosis in tissues, such as lung and bone, in which the Klotho protein is not expressed^14^. Meanwhile, CD9 and CD81, which are ubiquitously expressed in aged organs, function as molecular facilitators, or modulators of signal transduction pathways. These differences in localization and function could explain some of the phenotypic differences between these models, e.g., atherosclerosis in Klotho mice and cataracts in CD9/CD81 DKO mice. Because tetraspanins have never been reported to play a role in cataracts or atrophy of organs such as the testis, adipose tissue, thymus, pancreas, and submandibular glands, further studies are warranted. Neither decreased Klotho protein nor hypercalcemia was observed in the lung or epithelial cells of CD9/CD81 DKO mice; therefore, it is unlikely that the Klotho protein actively participates in the pathogenesis of DKO mice. Thirdly, while Klotho mice live for less than 2 months^14^, CD9/CD81 DKO mice live for approximately 1.5 years, indicating that the DKO mice should be viewed as undergoing accelerated senescence, rather than as manifesting a progeroid syndrome.

Importantly, it is unlikely that the emphysema in DKO mice resulted from defective alveolarization, because the lung phenotype in DKO mice is normal at the age of 3 weeks, by which time mouse alveolarization is complete^41^. Moreover, elastin/collagen staining and ultrastructural studies of histological sections suggested that the alveolar destruction and remodelling process were ongoing in the DKO lung^30^. Therefore, all of these findings in our previous papers could indicate that abnormal development had little, if any, effect in the lung pathology. Given that senile or aged lung is characterized by alveolar enlargement without wall destruction, a pathologic hallmark of emphysema^42^, our DKO mice could be regarded as a model of senile emphysema rather than merely a model of aged lung.

### Tetraspanins CD9/CD81 maintain the expression of SIRT1

Both COPD and aging are complex and highly heterogeneous conditions. Consequently, the molecular mechanisms that govern these processes remain incompletely understood. Importantly, Sirt1 overexpression attenuates the upregulation of the senescence markers p21, p16, and p53 in the lungs of SIRT1 heterozygous mice, thereby protecting against lung senescence in epithelial cells, but not in macrophages^23^.

In this study, deletion of CD9 and CD81 in epithelial cells additively downregulated the expression of SIRT1, thereby decreasing Foxo3a expression, while upregulating the expression of p53 and p21. Despite the similar localizations and functions of the two proteins, CD9 and CD81, and the similar phenotypes of the corresponding KO mice, it is not necessarily the case that these tetraspanins exert additive effects on all aspects of physiology^43^. For example, deletion of CD9 in epithelial cells moderately up-regulated expression of p21, but deletion of CD81 had a much more dramatic effect (Fig. 6a). Thus, each tetraspanin might exert additive effects on some processes, and distinct effects in others, thereby contributing to the mechanism of ageing (Supplemental Fig. 5). SIRT1720, a specific activator of SIRT1, rescues CS-induced emphysema in mice via upregulation of Foxo3a, a well-known SIRT1 target^20,44^. Consistent with this, in our model, SRT1720 rescued the diminished expression of Foxo3a, and reciprocally downregulated p21, suggesting that reduced SIRT1 expression is partially responsible for the age-related expression patterns of molecules such as Foxo3a and p21 (Supplemental Fig. 7). Furthermore, DNA microarray data from DKD epithelial cells were akin to those of DKO lungs, indicating that the biological processes active in epithelial cells might reflect those in the lung, at least to some extent (Supplemental Fig. 6). Although SIRT1 functions as a protein/histone deacetylase targeting many substrates, including p53 and NF-κB, the upregulation of p21, and p53 could not be entirely attributed to the reduced expression or activity of SIRT1. Alternatively, the upregulation of p21 and p53 might be partially due to the effect of senescence following deletion of CD9 and CD81 by different pathways to SIRT1. Moreover, we could not determine how CD9 and CD81 downregulate the expression of SIRT1, although bioinformatics analysis suggested that integrin α5β1, CD44, and CD38 might be involved (data not shown).

Notably, expression of SIRT6 is reduced in lung homogenates from COPD patients and SIRT6-regulated CSE-induced cell senescence in human bronchial epithelial cells (HBECs)^45^. In this study, however, SIRT6 expression was not altered *in vitro* or *in vivo*; therefore, it is unlikely that SIRT6 plays a key role in the pathogenesis of DKO mice (Fig. 5).

Another key aspect in aging is chronic inflammation (so-called ‘inflammaging’)^46^. This process is characterized by activation of several signaling molecules, including NF-κB, Forkhead box O, and Klotho. A reduction in SIRT1 expression is associated with elevated activation of RelA/p65 NF-κB, the master regulator of inflammation^22,47^ Importantly, CD9/CD81 DKO mice exhibited increased inflammation *in vitro* and *in vivo* at both the protein and mRNA levels (Figs 1, 2, 4, 7 and Supplemental Figs 5 and 6). Consistent with this, double deletion of CD9 and CD81 in the epithelial cells downregulated expression of SIRT1, thereby increasing the acetylation of NF-κB, indicating that the SIRT1 pathway might also be involved in persistent inflammation in this mouse model (Fig. 7). However, given that SIRT1 and other anti-aging proteins interact in a complex manner, and that tetraspanins are a multifunctional family of membrane organizers, alternative pathways could also be involved (Fig. 4a). Indeed, both CD9 and CD81 inhibit inflammation by modulating MMP-9 production and migration in macrophages, and CD9 KO mice exhibit elevated inflammation *in vitro* and *in vivo* after LPS stimulation, mediated by the disruption of complexes between tetraspanins and integrins or other binding partners^31,32^.

### COPD: from spill-over theory to disorder of molecular organization

Although the spill-over effect has been considered to be the major mechanism underlying the comorbidities of COPD, this theory is not sufficient to explain this complex systemic disease. Given their pleiotropic functions as molecular organizers and their ubiquitous expression, tetraspanins could affect not only the spill-over effect, but also exert local effects on several pathways, as seen in our bioinformatic analysis (Fig. 4 and Supplemental Figs 5 and 6). Although elevated inflammation and MMP production could affect some aging phenotypes through the spill-over effect in our model, several aging phenotypes could be explained without invoking the spill-over theory.

First, both CD9 and CD81, which are abundantly expressed in macrophages and myoblasts, regulate fusion and differentiation into osteoclast and myoblasts, respectively, both *in vitro* and *in vivo*^29,30,48^. Therefore, the osteopenia and sarcopenia seen in DKO mice cannot simply be attributed to the spill-over effect from the lung, but must involve disorganization of the fusion machinery after CD9/CD81 depletion. Second, both CD9 and CD81, which are also abundantly expressed in vascular endothelial cells and lymphatic endothelial cells, additively promote angiogenesis and lymphangiogenesis *in vitro* and *in vivo*^49^. Because VEGF signaling plays critical roles *in vivo* during the development of the early cardiovasculature and several organ systems^50^, a reduction of angiogenesis or possibly lymphangiogenesis might contribute to the atrophy of organs such as muscle and adipose tissue in DKO mice. Thus, CD9 and CD81 might regulate inflammation/COPD/aging not only through the spill-over effect, but also by organizing molecular complexes in tetraspanin-enriched microdomains. However, given their pleiotropic functions and their ubiquitous expression of tetraspanins, further study using a lung specific DKO mouse would be intriguing in order to verify the contribution of tetraspanins in the lung on extrapulmonary effect.

### Therapeutic applications for COPD and aging

Because COPD is increasingly regarded as a major health problem around the world, there is an urgent need to develop novel therapies for this disease. Given that anti-aging molecules have been developed with some efficacy in mouse and human, and that COPD has been viewed as a form of accelerated senescence, anti-aging molecules could be used to treat COPD. Given that SIRT1 functions as a hub protein among the complex networks associated with aging, and that tetraspanins protect against senescence through both direct and indirect interactions with SIRT1, pharmacological modulation of tetraspanins represents a novel therapeutic strategy for both COPD and aging^51,52^. Using a drug-repositioning strategy, we identified 72 drugs that upregulate CD9 expression from a library of 1,165 compounds. Among them, the statins exerted anti-inflammatory effects by upregulating tetraspanin CD9 and CD81 expression in macrophages^53^. Although statins possess potent anti-inflammatory properties that positively affect COPD and cardiovascular disease^54^, these beneficial effects might be attributable to the upregulated expression of CD9 and CD81. Recently, a structural study of full-length CD81 revealed that this protein has a cone-like architecture and undergoes a conformational change after cholesterol binding, suggesting a potential mechanism for regulation of tetraspanin function^55^.

Therefore, CD9/CD81 DKO mice represent a unique model for COPD leading to accelerated senescence. Furthermore, the molecular organizers CD9 and CD81, which might maintain the expression of SIRT1 as well as other aging pathways, are promising novel therapeutic targets, not only for COPD, but also for aging^56^.

## Electronic supplementary material

Supporting information

Supplemental Table 3

Supplemental Table 4
