# Supplementary material for: Double deletion of tetraspanins CD9 and CD81 in mice leads to a syndrome resembling accelerated aging
Source: Sci Rep. 2018 Mar 23;8:5145. doi: 10.1038/s41598-018-23338-x (PMC5865149; doi:10.1038/s41598-018-23338-x)
Supplement: Supplementary file 1 — Supporting information [file 41598_2018_23338_MOESM1_ESM.pdf]

Supporting Information

Double deletion of tetraspanins CD9 and CD81 in mice leads to a syndrome resembling accelerated aging

Yingji Jin[1#], Yoshito Takeda[1*#], Yasushi Kondo[2#], Lokesh P. Tripathi[3], Sujin Kang[1], Hikari Takeshita[4], Hanako Kuhara[1], Yohei Maeda[1], Masayoshi Higashiguchi[1], Kotaro Miyake[1], Osamu Morimura[1], Taro Koba[1], Yoshitomo Hayama[1], Shohei Koyama[1], Kaori Nakanishi[1], Takeo Iwasaki[1], Satoshi Tetsumoto[1], Kazuyuki Tsujino[1], Muneyoshi Kuroyama[1], Kota Iwahori[1], Haruhiko Hirata[1], Takayuki Takimoto[1], Mayumi Suzuki[1], Izumi Nagatomo[1], Ken Sugimoto[4], Yuta Fuji[2], Hiroshi Kida[1], Kenji Mizuguchi[3], Mari Ito[3], Takashi Kijima[1], Hiromi Rakugi[4], Eisuke Mekada[5], Isao Tachibana[1], Atsushi Kumanogoh[1]

[1] Department of Respiratory Medicine and Clinical Immunology, Osaka University Graduate School of Medicine, Suita, Osaka, Japan, [2]Sumitomo Dainippon Pharma Co., Ltd, Osaka, [3]National Institute of Biomedical Innovation, Health and Nutrition, Osaka, [4]Department of Geriatric Medicine &, Osaka University Graduate School of Medicine, [5]Department of Cell Biology, Research Institute for Microbial Diseases, Osaka University

SUPPLEMENTAL METHODS

Transmission electron microscopy

Tissue samples were fixed at 4 ºC in 2.5% glutaraldehyde in 0.1 M phosphate buffer (pH 7.2) and post-fixed at room temperature in 2% osmium tetroxide in the same buffer. After dehydration in ethanol, the specimens were embedded in Epon 812 resin (TAAB, Berkshire, UK). Ultrathin sections (60 nm) were stained with uranyl acetate and lead citrate, and observed under a transmission electron microscope (H-7100; Hitachi, Tokyo, Japan)

Lung function test

Lung volume and compliance were measured with a whole-body plethysmograph as part of the Pulmonary Maneuvers System (Buxco Electronics, Wilmington, NC). Anesthetized mice were attached via a tracheostomy tube to a mechanical ventilator in a sealed plethysmograph. A quasi-static pressure–volume maneuver was used to measure lung compliance. Data for all maneuvers were analyzed with Biosystem XA software (Buxco Electronics).

Grip test and spontaneous locomotor activity

Forearm grip strength was assessed 3 times in 8-wk-old mice using a grip strength meter (GPM-100; Melquest, Toyama, Japan) according to the manufacturer's instructions (1). Spontaneous locomotor activities of test and control mice were measured simultaneously in separate transparent-plastic cages using a LOCOMO sensor system consisting of LOCOMO sensor units (LS-5), a counter interface (CIF-mini4), a control unit (LCU-20), and a personal computer with WinCIF II mini software (Melquest). The counter interface counted the interruptions of latticed infrared-beams by a mouse moving in the cage inside the sensor.

Micro-computed tomography (CT)

The mice were anesthetized with isoflurane and placed in the chamber of a CT scanner for small animals (Latheta LCT-200; Hitachi, Tokyo, Japan) (2). Bone mineral density and the muscle mass were assessed by analyzing the volume using the tibia. Visceral adipose tissue was analyzed by assessing fat content within the peritoneal cavity. Three-dimensional CT pictures for whole body were reconstructed with VGStudio MAX2.0 software (Volume Graphics, Tokyo, Japan).

Immunohistochemistry

Mice paraffin sections (WT and DKO) were stained using indirect immunohistochemistry as described previously with minor modifications (3). Briefly, deparaffinized and rehydrated tissue sections were heated in citrate buffer (15 min, at 121°C, 10 mM, pH 6.0), and blocked with 10% normal serum. Sections were incubated with rabbit anti-p21 monoclonal antibody (EPR18021; dilution 1:200, Abcam), overnight at 4°C, and subsequently incubated with biotin anti-rabbit immunoglobulin antibody (dilution 1:100; Vector Laboratories, Burlingame, CA) for 20 min. Signals were visualized using avidin-biotin peroxidase (Vectastain Elite kit) with 3,3'-diamino-benzidine (DAB; Sigma) and counterstained with

Nuclear Fast Red (Vector Laboratories). A minimum of three fields from each mouse lung (n=3) were obtained at x 200 magnification and the p21-positive cells per field were quantitated.

Bioinformatics

IPA(Ingenuity Systems; www.ingenuity.com) is a knowledge base that contains approximately 3,000,000 biological and chemical interactions and functional annotations with definite scientific evidence. By uploading the list of Gene IDs, the network-generation algorithm identifies genes integrated in a global molecular network. IPA calculates a *P*-value score that reflects the statistical significance of the association between the genes and the networks by Fisher's exact test. Array data were analyzed using IPA, comparing the gene expression lists of 15W CD9/CD81 DKO lungs and WT lungs; significantly differentially regulated genes were classified into subcategories by gene functions.
TargetMine(http://targetmine.nibio.go.jp) is an integrative data analysis platform for gene set analysis and knowledge discovery within the framework of a data warehouse. TargetMine models biological entities such as genes and proteins as "objects" and their relationships as "references" to other objects. TargetMine features a variety of biological data types and interactive tools to analyse them. In this study, biological pathway data from KEGG and Gene Ontology (GO) Slim associations from the GO consortium were used to assign functional annotations to the differentially expressed genes (DEGs) in 12W CD9/CD81 DKO (and their human orthologues) and in CD9/CD81 DKD epithelial cells relative to the WT. To better understand the functional implications of the differential gene expression levels in the context of the human lung, we used the one-click gene conversion function within the lists page of TargetMine obtain human orthologs of the DEGs in the 12W CD9/CD81 DKO. The enrichment of specific KEGG pathways and GO Slim (biological process) term associations within each DEG set was estimated by performing Fisher's exact test within TargetMine. The inferred p-values were further adjusted for multiple test correction to control the false discovery rate using the Benjamini and Hochberg procedure and the annotations were considered significant if the adjusted p-value was 0.05 or less.

KeyMolnet (KeyMolnet Data; www.km-data.jp/keymolnet) contains knowledge-based information on 164,000 relationships among human genes and proteins, small molecules, diseases, pathways, and drugs.21 It includes core content collected from selected review articles with the highest reliability. By importing the list of Gene IDs, KeyMolnet automatically provides corresponding molecules as nodes on the network. The neighboring network-search algorithm selects one or more molecules as starting points to generate a network of all kinds of molecular interactions around the starting molecules, including direct activation/inactivation, transcriptional activation/repression, and complex formation within one path from the starting points. The generated network is compared side by side with 501 human canonical pathways of the KeyMolnet library. The algorithm counting the number of overlapping molecular relationships between the extracted network and the canonical pathway makes it possible to identify the canonical pathway showing the most significant contribution to the extracted network.

Supplemental Table 1. Histological analysis of aging phenotype in CD9/CD81 DKO mice.

Organ		Pathology	WT			DKO		
			1	2	3	1	2	3
Eye		cataract	+	+	+	2+	2+	2+
Thymus		atrophy	−	−	−	2+	−	2+
Spleen		atrophy	−	−	/	+	+	±
Pancreas		acinar atrophy	±	−	/	+	+	+
		atrophy islands of Langerhans	−	−	/	+	+	+
Heart		thickening of arterial wall	+	2+	−	−	−	−
Aorta		arteriosclerosis	−	−	−	−	−	−
Skin		decrease of adipose tissue	−	−	/	2+	2+	2+
		decrease of hair follicle	−	−	−	+	+	+
Femur		thinning of cortical bone	−	−	−	+	±	+
		thinning of epiphyseal cartilage	−	−	−	+	±	+
		decrease of trabecular bone	−	−	−	+	±	+
Thigh muscle		atrophy	−	−	−	2+	2+	2+
Fatty tissue		atrophy	−	−	/	2+	+	+
Testis		atrophy	−	−	/	2+	2+	−
Vesicula seminalis		atrophy	−	−	−	+	+	/
Prostate gland		atrophy	−	−	−	+	+	/
Ovary		atrophy	+	+	±	±	±	−
Brain	Cerebrum	vacuolation	−	−	−	+	+	+
		amyloid (Congo red)	−	−		−	−	
	Pituitary	Atrophy	−	−	−	+	+	+
Submandibular gland		loss of granules in the striated portion of the epithelium	−	−	−	2+	−	2+
Liver		cell infiltration	±	−	±	±	−	±
Kidney		cell infiltration	±	+	+	+	±	±
Gastrointestinal tract		cell infiltration	−	−		−	−	

Supplemental Table 1. Histological analysis of aging phenotype in CD9/CD81 DKO mice.

Shown are tables regarding histological analysis of aged mice from WT type (left) and DKO mice (right) (n=2-3, 18 months of age). Pathological scoring is reported as negative (−), mild (±), moderate (+) and severe (2+) by experts in animal pathology. Multiple organ atrophy was confirmed in aged DKO mice. However, no significant difference was observed in several organs such as heart, kidney, liver, gastrointestinal tract and aorta.

Supplemental Table 2. Hematological and serological analyses of CD9/CD81 DKO mice.

Test item		Wild-type (n = 6)	DKO (n = 3)	*P*-value
**Complete Blood Count**				
White Blood Cells	($\times 10^9$, /L)	3.33 ± 1.49	7.04 ± 6.59	0.432
Lymphocytes	($\times 10^9$, /L)	2.79 ± 1.17	6.11 ± 6.39	0.463
Monocytes	($\times 10^9$, /L)	0.15 ± 0.06	0.12 ± 0.12	0.710
Granulocytes	($\times 10^9$, /L)	0.39 ± 0.39	0.81 ± 0.45	0.249
Red Blood Cells	($\times 10^{12}$, /L)	10.4 ± 1.13	10.7 ± 1.71	0.789
Hemoglobin	(g/dL)	13.0 ± 1.58	11.3 ± 2.05	0.299
Platelets	($\times 10^{12}$, /L)	0.85 ± 0.15	1.07 ± 0.04	0.015**
**Blood Biochemistry**				
Total protein	(g/dL)	5.52 ± 0.12	4.53 ± 0.46	0.062
Albumin (BCG)	(g/dL)	3.38 ± 0.21	2.97 ± 0.40	0.208
Triglycerides	(mg/dL)	116 ± 44.3	75.3 ± 45.7	0.276
Total cholesterol	(mg/dL)	113 ± 7.81	90.3 ± 4.93	0.0015**
AST	(IU/L)	58.3 ± 11.9	86.7 ± 18.9	0.104
ALT	(IU/L)	33.5 ± 19.7	38.7 ± 10.4	0.623
Creatinine	(mg/dL)	0.10 ± 0.02	0.11 ± 0.04	0.585
Calcium	(mg/dL)	10.1 ± 0.25	9.17 ± 0.15	0.0003**
Inorganic phosphorus	(mg/dL)	10.5 ± 1.47	10.8 ± 1.87	0.832

Supplemental Table 2. Hematological and serological analyses of CD9/CD81 DKO mice.

By hematological and serological test, level of creatinine and phosphate was indistinguishable, whereas that of cholesterol and calcium was minimally decreased, and that of platelet was increased. ** $P < 0.01$ versus WT.

Reference

1. Nakae Y., Quantitative evaluation of the beneficial effects in the mdx mouse of epigallocatechin gallate, an antioxidant polyphenol from green tea. *Histochem Cell Biol.* **137,** 811-827 (2012)

2. Nakanishi K., *et al.* Involvement of Endothelial Apoptosis Underlying Chronic Obstructive Pulmonary Disease-like Phenotype in Adiponectin-null Mice: Implications for Therapy. *Am J Respir Crit Care Med.* **183,** 1164-1175 (2011)

3. Tsujino K. *et al.* Tetraspanin CD151 protects against pulmonary fibrosis by maintaining epithelial integrity. *Am J Respir Crit Care Med.* **186,** 170-180 (2012)

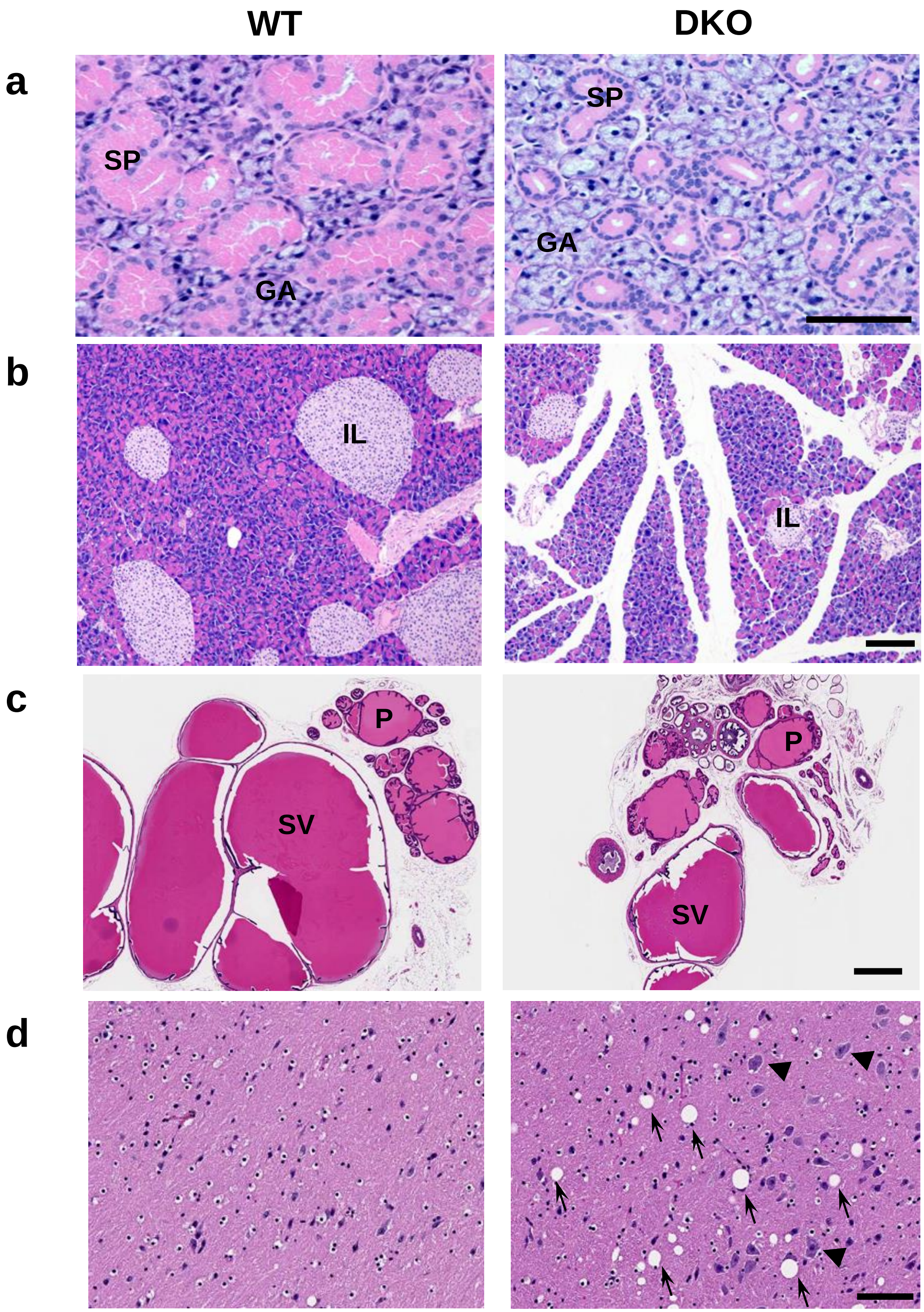

Supplemental Figure 1. Histological analysis of aging phenotype in CD9/CD81 DKO mice.
(a-d) Histological sections were from submandibular glands/striated portion (SP)/glandular acinus (GA) (a), pancreas/islands of Langerhans (IL) (b), seminal vesicles (SV) / prostate (P) (c), and cerebrum (d). Note that submandibular glands, pancreas, and seminal vesicles/ prostate from DKO mice were markedly atrophic. While no histological difference could be observed in the ovary, atrophy was remarkable in the testis of DKO mice. IL; islet of Langerhans. (d) The number of vacuoles and enlarged glial cells could be increased in aged DKO mice. (Arrow; vacuoles, Arrowhead; enlarged cell) Data are representative of three independent studies with similar results. Scale bar, 100 μm for (a), (b), (d) and 1 mm for (c).

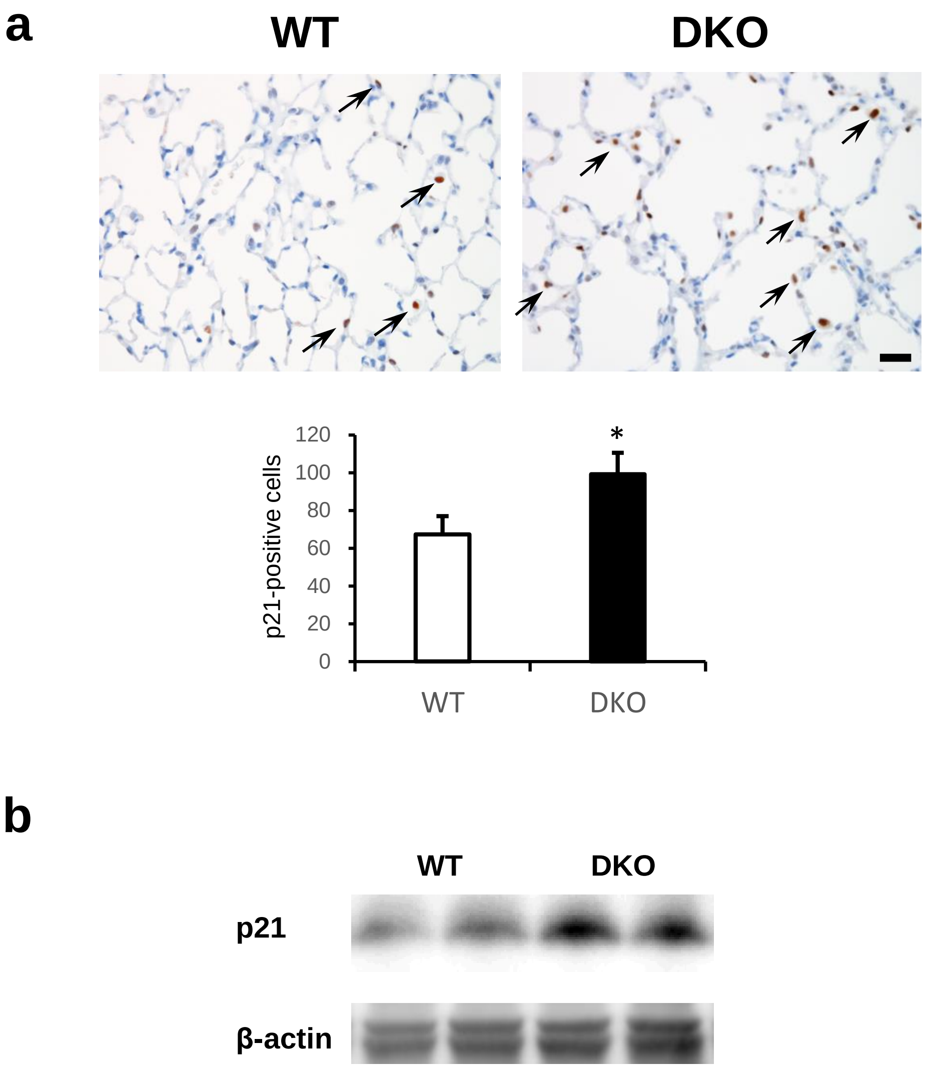

Supplemental Figure 2. p21-positive cells in lungs of CD9/CD81 DKO mice.

(a) Immunostaining and (b) Immunoblot for p21 of WT and the DKO lungs. Notably, p21-positive cells (arrows) were increased in the DKO lungs in compared with those in the WT lungs at 18 months of age. Bars represent means ± SD; * $P < 0.05$ versus WT. Scale bar, 20 μm for (a)

a

Month		6						18					
Mouse		WT1	WT2	WT3	DKO1	DKO2	DKO3	WT1	WT2	WT3	DKO1	DKO2	DKO3
Behaviour	reactivity	0	0	0	0	0	0	0	0	1	1	2	2
	passivity	0	0	0	0	0	0	1	0	0	1	2	2
Appearance (skin & hair)	glossiness	0	1	0	1	1	1	1	1	1	2	1	2
	coarseness	0	0	0	0	0	0	0	1	1	1	1	1
	loss of hair	0	0	0	0	0	0	0	0	0	1	1	2
	skin ulcers	0	0	0	0	0	0	0	0	0	0	0	0
Eyes	catarrhal change	0	0	0	0	0	0	0	0	0	1	1	1
Spine	kyphosis	0	0	0	1	1	2	0	1	1	2	3	3

b

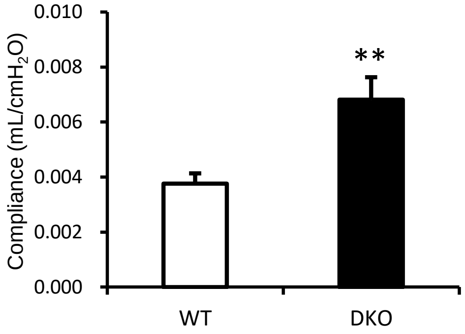

c

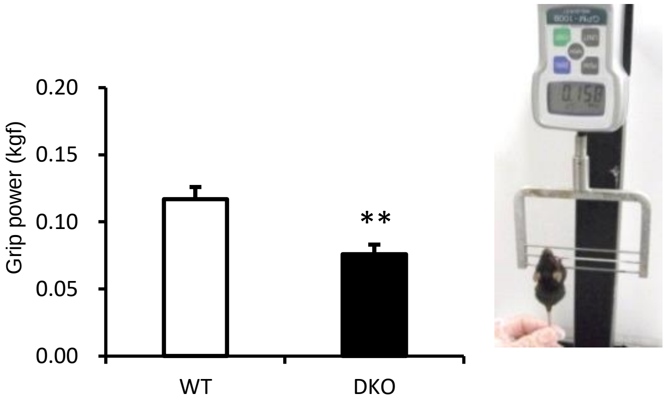

Supplemental Figure 3. Functional aging in CD9/CD81 DKO mice.
(a) The degree of aging were evaluated according to the grading score of aging in mice (n=3). No aging phenotype was detected at 1 month of age. (b and c) DKO mice not only showed the decreased respiratory function (n=5), but also decreased grasping power (n=3-4). Bars represent the mean ± SD; ** P < 0.01 versus WT.

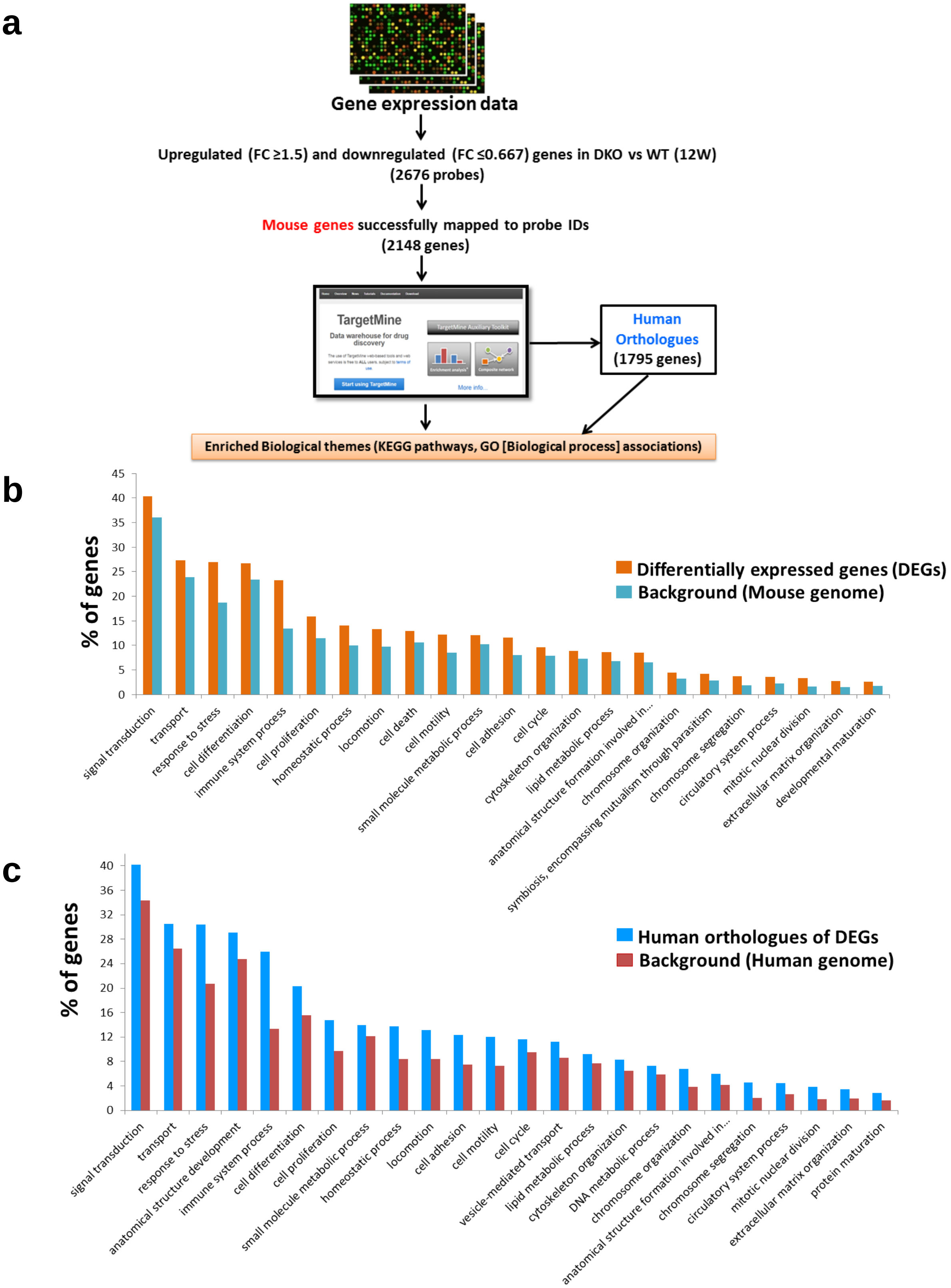

Supplemental Figure 4. DNA microarray analysis of CD9/CD81 DKO mice using TargetMine.

(a) The list of genes differentially expressed between WT and DKO lungs (n=1) were analyzed using TargetMine, and they were converted to human orthologues. (b) The gene categories with differentially expressed in DKO lungs from Enriched Gene Ontology (GO) Slim [Biological Process] ($p \leq 0.05$) are consistent with IPA results, in which, cell death, proliferation, and inflammation were highly ranked. (c) The multiple aging phenotypes in DKO mice were further supported by the Enriched Gene Ontology (GO) Slim [Biological Process] ($p \leq 0.05$) in human orthologues.

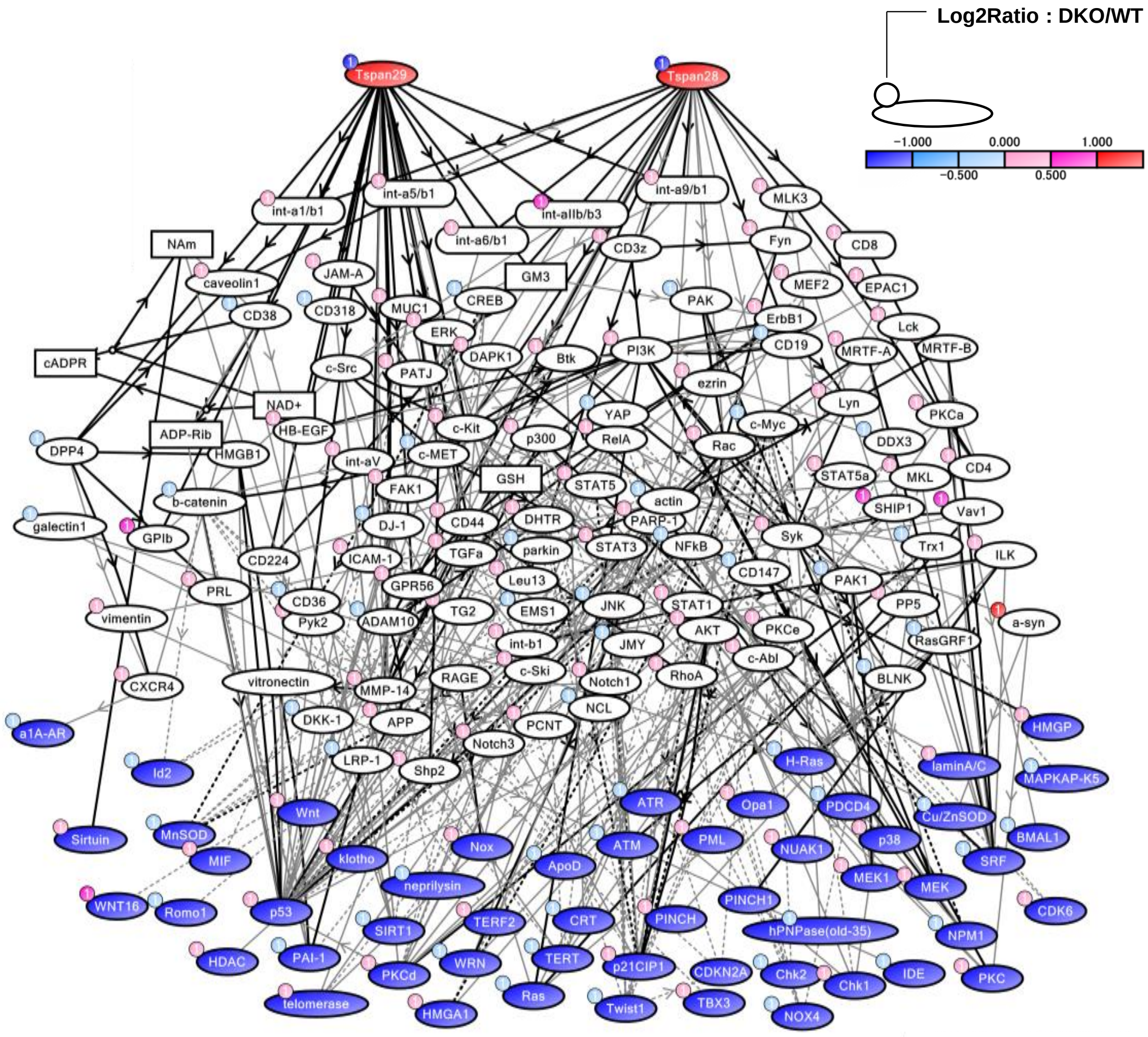

Supplemental Figure 5. Comprehensive DNA microarray analysis of the lung.

KeyMolnet software revealed the network of relevant regulatory relationships between tetraspanins (CD9 and CD81) and their downstream pathways related to aging. TSPAN29: CD9, TSPAN28: CD81.

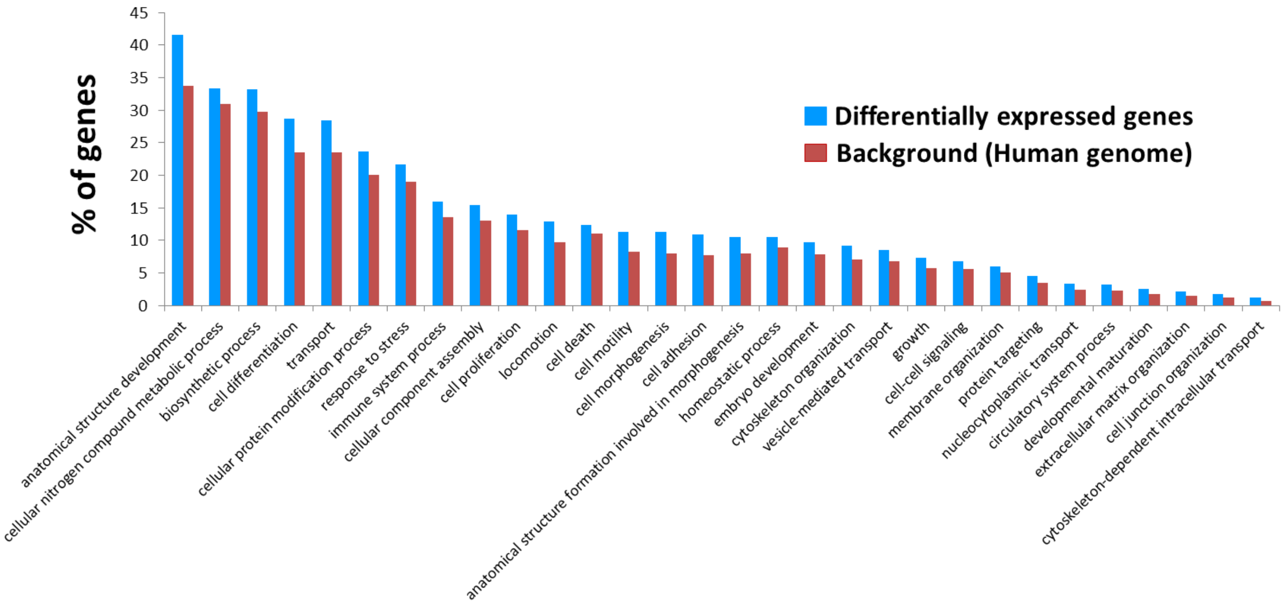

Supplemental Figure 6. DNA microarray analysis of CD9/CD81 DKD epithelial cells using TargetMine.

DNA microarray analysis of CD9/CD81 DKD epithelial cells (n=3) by TargetMine demonstrated Enriched Gene Ontology Slim (biological process). Consistent with IPA results, cell death, proliferation, and inflammation were highly ranked.

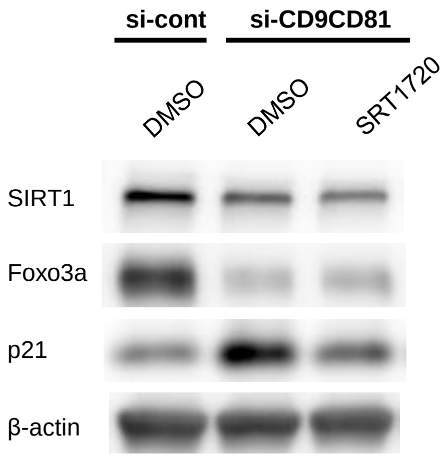

Supplemental Figure 7. SIRT1 activator rescued the senescence in CD9/CD81 DKD epithelial cells. SRT1720 partially rescued p21 increase as well as Foxo3a reduction by DKD of CD9 and CD81.
